# Anabolic Strategies for ICU-Acquired Weakness. What Can We Learn from Bodybuilders?

**DOI:** 10.3390/nu16132011

**Published:** 2024-06-25

**Authors:** Jakub Tarnawski, Maja Czub, Marta Dymecki, Medha Sunil, Marcin Folwarski

**Affiliations:** 1 Medical University of Gdansk, 80-210 Gdańsk, Poland; 2 Department of Endocrinology and Internal Diseases, Medical University of Gdansk, 80-210 Gdańsk, Poland; 3 Independent Public Health Care Center, Ministry of Internal Affairs and Administration, 80-104 Gdańsk, Poland; 4 Students’ Scientific Circle of Clinical Nutrition, Medical University of Gdansk, 80-210 Gdańsk, Poland; 5 Department of Clinical Nutrition and Dietetics, Medical University of Gdansk, 80-210 Gdańsk, Poland; 6 Home Enteral and Parenteral Nutrition Unit, General Surgery, Nicolaus Copernicus Hospital, 80-803 Gdansk, Poland

**Keywords:** intensive care unit-acquired weakness, nutrition, ICU, anabolism, muscles

## Abstract

The study aimed to show the potential clinical application of supplements used among sportsmen for patients suffering from Intensive Care Unit-acquired Weakness (ICUAW) treatment. ICUAW is a common complication affecting approximately 40% of critically ill patients, often leading to long-term functional disability. ICUAW comprises critical illness polyneuropathy, critical illness myopathy, or a combination of both, such as critical illness polyneuromyopathy. Muscle degeneration begins shortly after the initiation of mechanical ventilation and persists post-ICU discharge until proteolysis and autophagy processes normalize. Several factors, including prolonged bedrest and muscle electrical silencing, contribute to muscle weakness, resulting from an imbalance between protein degradation and synthesis. ICUAW is associated with tissue hypoxia, oxidative stress, insulin resistance, reduced glucose uptake, lower adenosine triphosphate (ATP) formation, mitochondrial dysfunction, and increased free-radical production. Several well-studied dietary supplements and pharmaceuticals commonly used by athletes are proven to prevent the aforementioned mechanisms or aid in muscle building, regeneration, and maintenance. While there is no standardized treatment to prevent the occurrence of ICUAW, nutritional interventions have demonstrated the potential for its mitigation. The use of ergogenic substances, popular among muscle-building sociates, may offer potential benefits in preventing muscle loss and aiding recovery based on their work mechanisms.

## 1. Introduction

The investigation of nutritional tactics sourced from athletes and bodybuilders has captured our interest due to its potential applicability in ICUAW treatment. Among the various supplements studied, creatine and whey have gained prominence as extensively researched gym supplements known for their ability to promote muscle growth and alleviate muscle damage. Moreover, emerging findings suggest the potential of HMB supplementation in safeguarding and enhancing lean body muscle mass, while vitamin D supplementation shows promise, particularly in individuals with severely low initial levels. Additionally, the consideration of anabolic-androgenic steroids (AASs) may be justified in specific scenarios, given their potential benefits, particularly in addressing hormonal imbalances associated with conditions like sarcopenia. Furthermore, the exploration of multimodal therapy, including the utilization of insulin-like growth factor 1 (IGF-1), presents promising avenues for alleviating catabolic states and musculoskeletal impairment. Alongside nutrition, physical rehabilitation emerges as a crucial aspect of the recovery process, with early mobilization and resistance exercises indicating the potential to reverse muscle loss and prevent further deterioration. As we delve deeper into the potential advantages of integrating supplementing utilized by athletes into the recovery journey of individuals grappling with critical illness, an exciting prospect emerges for enhancing outcomes and bolstering ICU patients. This paper endeavors to illuminate the potential of athlete-derived nutritional strategies in optimizing the recovery trajectory of individuals confronting intensive care unit-acquired weakness.

### Materials and Methods

We searched the MEDLINE and SCOPUS databases using the terms “ICU-acquired weakness”, “nutritional interventions”, “anabolic strategies”, “muscle degeneration”, “proteolysis”, “creatine supplementation”, “whey protein”, “β-Hydroxy-β-methylbutyrate (HMB)”, “vitamin D”, “anabolic-androgenic steroids”, “testosterone therapy”, “growth hormone”, “Insulin Growth Factor 1”, “beta-blockers”. The search primarily focused on studies published in the last 10 years.

## 2. In-Depth Review and Insights

### 2.1. Definition, Diagnosis, and Consequences

Intensive Care Unit-acquired weakness occurs in approximately 40% of critically ill patients [1] and is a long-term complication of ICU admission that may lead to functional disability [2]. The whole process consists of critical illness polyneuropathy, critical illness myopathy, or both of them, like critical illness polyneuromyopathy [3,4]. Muscle degeneration starts within hours after the initiation of mechanical ventilation [5] and continues post-ICU discharge until proteolysis and autophagy are normalized [6].

Currently, there is no reference standard for ICUAW diagnosis, but the American Thoracic Society guidelines recommend using the Medical Research Council (MRC) muscle strength score. The result of an average MRC muscle strength score lower than 4 across all determined by manual muscle testing (MMT), without an alternative explanation of muscle weakness to critical illness, suggests ICUAW [7]. As ultrasound is nowadays easily accessible in ICU, quadriceps muscle, especially rectus femoris and vastus lateralis, ultrasound is being evaluated as a noninvasive tool for muscle atrophy identification [8]. Additionally, among the patients without acute kidney injury (AKI), the sarcopenia index, considered as serum creatinine-to-serum cystatin C ratio, might be helpful in muscle mass estimation and following its changes [9].

ICUAW is recognized as a predictor of short-term and long-term consequences [10]. Patients with ICUAW are more likely to develop swallowing disorders [11], experience extubation failure [12], and need prolonged mechanical ventilation and longer ICU admission, which increases healthcare-related costs [13,14]. Moreover, the persistence of muscle weakness at ICU discharge raises the 1-year mortality ratio [14].

The pandemic of Severe Acute Respiratory Syndrome Coronavirus (SARS-CoV-2), resulting in a plethora of patients with a need for prolonged sedation and ventilation, provided a deeper insight into ICUAW consequences [15]. While the mortality rate among SARS-CoV-2 patients seems to be independent of muscle weakness, patients diagnosed with ICUAW stay longer in the ICU and have lower mobility scores at ICU discharge [16].

### 2.2. Pathomechanisms

There are several muscle weakness triggers, including prolonged bedrest and electrical silencing of the muscle [6]. Muscle wasting is generally caused by the imbalance between protein degeneration and/or its synthesis [6], among which atrophy of type-II myofibers and myosin heavy chain (MyHC) loss are consistently observed [2]. Although some molecular mechanisms are still unclear, certain processes contribute to muscle degeneration. These include increased Ca^2+^-dependent proteolysis, activation of the ubiquitin-proteasome system, and disrupted autophagy, all driven by inflammation, sepsis, and pro-inflammatory cytokines [4]. Respectively, protein synthesis is impaired due to blunted anabolic signaling and altered transcriptional regulation [4].

**Figure 1:**
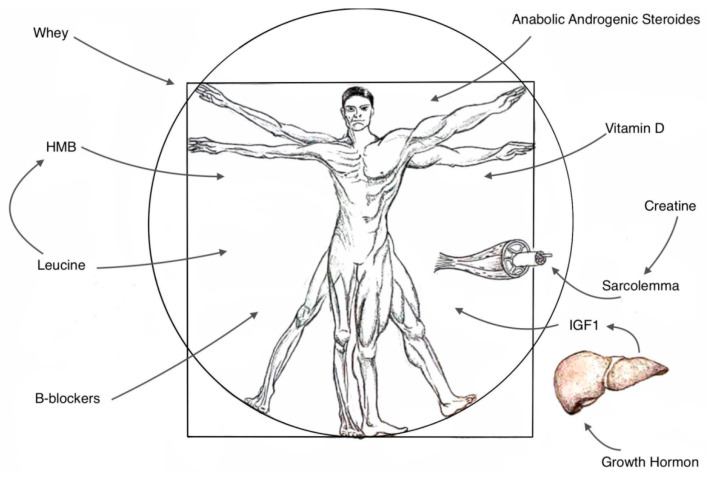
The original figure describes anabolic support in ICUAW.

Critical illness or sepsis, often connected with ICUAW, also causes a combination of factors like tissue hypoxia, increased oxidative stress, and insulin resistance, which lead to reduced glucose uptake, lower adenosine triphosphate (ATP) formation, mitochondrial dysfunction, and high free-radical production [17]. The indication of biomarkers connected with ICUAW might significantly help in the introduction of targeted therapy. They include creatine kinase, interleukin 1 (IL-1), interleukin 6 (IL-6), tumor necrosis factor-alpha (TNF-α), growth differentiation factor 15 (GDF-15), or plasma levels of neurofilaments [4,18,19] [Figure 1].

### 2.3. Strategies and Recommendations

Providing effective nutrition therapy for critically ill patients is challenging. Numerous trials have improved the understanding of patient needs in the ICU. The European Society for Clinical Nutrition and Metabolism (ESPEN) recommends incorporating physical activity into nutritional therapy for improved outcomes, delivering 1.3 g of protein equivalents per kg of body weight per day, and supplementation of vitamin D3 (500,000 UI) within a week for critically ill patients with low plasma levels (25-hydroxy-vitamin D < 12.5 ng/mL or 50 nmol/L) [20]. Reducing the risk of ICU complications and post-ICU muscle wasting can be best achieved by minimizing the chances of a complicated hospital stay and ICU admission through multimodal prehabilitation [21]. ESPEN advocates for preoperative nutritional support for patients who are malnourished or at risk of malnutrition [22]. Providing protein through oral nutritional supplements for patients undergoing major surgery can improve outcomes and prevent ICU admissions. This is particularly evident in elderly populations [23] and those undergoing major abdominal surgery [24].

There are currently no fully effective interventions that can reverse ICUAW; hence, understanding the pathomechanisms remains an important area of focus. However, preventative measures have had more success. For example, ensuring normoglycemia in ICU patients reduces the risk of developing critical illness polyneuropathy (CIP) [25]. Targeting dysregulated mechanisms in critical illness myopathy (CIM), like autophagy, may be a target for potential treatment. Metformin could potentially be used to induce autophagy by activating 5′AMP-activated protein kinase (AMPK), inhibiting mTOR, and reducing muscle wasting in CIM [15].

Insulin downregulates Ubiquitin–proteasome system (UPS)-mediated proteolysis, leading to autophagy and inducing protein synthesis; therefore, it can potentially treat autophagy. Studies have shown that intensive insulin treatment in critically ill patients improves outcomes by reducing the probability of developing CIP and CIM [26] and improving morbidity and mortality [27]. In brain-injured ICU patients, intensive insulin therapy has been shown to protect peripheral and central nervous systems and improve outcomes [25].

Muscle atrophy is one of the major components of muscle weakness and significant focus has been made to mitigate it nutritionally by supplementing with high amounts of protein early to offset the caloric deficit. Administration of parenteral nutrition to meet the caloric targets not met by enteral nutrition early on (within 48 h) during ICU admission impaired recovery, increased complications, and dependency on mechanical ventilation, thereby increasing time spent in the ICU [28,29,30]. Early administration of amino acids via parenteral nutrition instead of glucose and lipids to meet the caloric deficits did not reduce muscle atrophy. Delaying parenteral nutrition until the first week of ICU admission has been shown to promote rapid recovery with fewer complications [28,29].

Since immobility increases the risk of ICUAW, early mobilization and rehabilitation could decrease the burden of ICUAW. This would help with muscle protein synthesis and inhibit the catabolic pathway. Heavy sedation is often an obstacle while trying to enforce such interventions, and daily interruption of sedative infusions in critically ill patients undergoing mechanical ventilation reduces ventilation dependency and the length of ICU stay [31]. In patients where active mobilization and rehabilitation are not feasible or patients who are not compliant, neuromuscular electrical stimulation [32] (NMES), along with in-bed cycle ergometry, have shown results that warrant further research with larger trials [33]. Worth-mentioning support is ergogenic supplements, that have been tested in athletes and bodybuilders, also post injuries [34]. Studies evaluating the profit of those supplements in preventing muscle loss are limited; however, ergogenic aids may have possible utility in the recovery process [35]. Nutritional strategies and training regimens for athletes are designed to enhance muscle mass and functionality, as well as to modify cellular metabolism for improved athletic performance. Athletes often employ a variety of supplements and nutrition plans, some of which are supported by scientific evidence. In our study, we aim to address several of these strategies to describe the potential benefit of the most popular supplements alongside pharmaceuticals frequently used in the muscle-building process.

### 2.4. Creatine

Creatine is claimed to be one of the more extensively studied dietary supplements [36]. Physiologically, it is produced endogenously at an amount of about 1 g/d depending on the amount of meat in the diet [37,38]. It is widely reported that creatine has many positive features among sportsmen, such as amplifying the effects of resistance training—strength and hypertrophy [39,40,41]—and improving the results of high-intensity intermittent speed training and aerobic endurance in trials lasting more than 150 s [41,42]. Creatine has also been proven to have positive effects on strength. Numerous studies described an increase in overall body weight of 1–2 kg during the first week of loading as well as fat-free mass growth, even daily living performance, or neurological functionality in young or older people [43,44]. Creatine might also reduce post-exercise muscle damage via mechanisms stabilizing the sarcolemma [45].

The literature described previously that it can be potentially effective to use creatine for muscle degeneration prevention among people with sarcopenia. Based on a comparison of pathomechanisms and biomarkers between sarcopenia and ICUAW, creatine supplementation combined with proper exercises may play a significant role in supporting muscle mass and strength regain, although it is unclear whether it could prevent muscle loss associated with ICUAW [35].

There is a theory that sarcopenia is caused by atrophy and loss of skeletal muscle fibers, especially type II. The biochemical mechanism of sarcopenia is based on the imbalance between protein synthesis and degradation rates [46]. TNF-alpha, IL-1, IL-6, and oxidative damage agents, including oxLDL (oxidized low-density lipoprotein), are biological markers associated with sarcopenia [47,48,49,50]. Creatine metabolism makes it possible to counter muscle atrophy connected with sarcopenia directly by multiple anabolic and anti-catabolic pathways [51]. In the literature, it was also presented that creatine might have an antioxidant character, first reported by Matthews et al. [52]. The mechanism of this feature is yet not fully known, however, the raised activity of antioxidant enzymes or elimination of reactive oxygen species (ROS) and reactive nitrogen species (RNS) were reported [53].

Dosing of creatine consists of a loading phase with 0.3 g × (kg × 0.1) × (d × 0.1) for 5 to 7 days and a maintenance phase with 0.03 g × (kg × 0.1) × (d × 0.1) for 4–6 weeks. However, loading doses is not necessary [54]. Nowadays, after over 20 years of research, no adverse effect from recommended dosages of creatine supplementation on kidney health has been demonstrated [36]. To evaluate the potential of creatine supplementation in post-ICU patients, well-designed studies replicating strategies used in injured athletes may be useful [35].

### 2.5. Whey

Whey protein, a by-product of dairy processing, is among the most widely utilized supplements by athletes. It promotes myofibrillar protein synthesis (MPS) which leads to building and maintaining muscle mass. Overall daily protein intake in the range of 1.4–2.0 g protein/kg body weight/day is stated by the International Society of Sports Nutrition (ISSN) to be an optimal amount for sportsmen. It is rich in leucine and other branched-chain amino acids [55,56].

Data show that a 28-day mortality reduction of up to 50% was achieved when both energy and protein targets were reached in mechanically ventilated patients, in comparison to only proper energy intake [57]. ESPEN guidelines from August 2018 recommend 1.3 g of protein equivalents per kg of body weight per day for ICU patients [20]. However, during the chronic ICU phase and after ICU discharge, higher targets should be provided. After discharge dose should be up to 1.5–2 g of protein/kg/day. During the convalescence period, 2–2.5 g of protein/kg/day for a prolonged duration is necessary to optimize the outcome. It should be kept in mind that patients who require respiratory therapy are at risk of low protein intake after extubation [58].

Unfortunately, many ICU patients receive calorie-rich but protein-deficient nutritional support, with even less than half of the recommended protein intake [59,60]. Leucine-rich protein beverages, such as whey protein shakes, are specifically beneficial during fasting-induced catabolic conditions [61]. Moreover, whey has a relatively good safety profile. It is widely used, without any noticeable side effects, by athletes, but also in older adults, a 6-month intervention of up to 2 servings (21 g protein, 3 g leucine per serving) did not result in kidney function deterioration [62,63]. As shown in the systematic review, adverse effects depend on many aspects, but the profit-to-loss ratio seems to be beneficial [64]. Due to its availability and cost-effectiveness, whey protein supplementation may be profitable both for critically ill patients during catabolism and post-ICU [35].

### 2.6. β-Hydroxy-β-Methylbutyrate (HMB)

HMB is produced endogenously as a leucine metabolite. As a supplement, HMB may affect inflammatory cascades and, due to the anabolic effect (mTOR pathway), modulate protein metabolism [65]. So far, many studies have shown that HMB supplementation can increase lean body mass with reduced markers of muscle damage [66]. HMB supplements have been proven to preserve muscle mass during 10 days of bed rest in older people [67]. Moreover, in a multi-center trial, a diet enriched in HMB improved muscle mass, prevented the onset of sarcopenia, and was associated with functional improvement in hospitalized older adults [68]. Although it is not clear whether HMB can prevent muscle loss among ICU-admitted patients [69], some authors suggest that it could benefit patients’ outcomes and recovery after a critical illness [70]. Because usually HMB intervention is connected with other supplementation, data on the direct impact of HMB alone are limited, and more studies examining the effect of HMB administrated alone are needed to obtain the conclusion.

### 2.7. Vitamin D

Vitamin D is a group of steroid compounds demonstrating the same biological activity. Its forms that occur in nature are vitamin D2 (ergocalciferol), known as the plant form of vitamin D, and vitamin D3 (cholecalciferol), which is produced in the skin of many vertebrate animals, including humans, by UVB exposure from 7-dehydrocholesterol (provitamin D3) [71]. The liver metabolizes either of them to 25(OH)D, which is further hydroxylated to the active form 1,25(OH)2D in the kidneys.

Due to common vitamin D deficiency in the population, many studies have explored the correlation between low vitamin D levels and impaired sports performance, defined as skeletal muscle function. The correlation between low circulating levels of vitamin D and muscle metabolism disorders is documented in various contexts, including muscle recovery, atrophy, sarcopenia, and cachexia. Therefore, the interaction between vitamin D deficiency and skeletal muscle atrophy signaling pathways has been studied in the last few years. Vitamin D deficiency is associated with oxidative stress in skeletal muscle that induces mitochondrial dysfunction and, through a possible signaling pathway triggering the expression of atrogin-1, which causes protein degradation, may contribute to the development of muscle atrophy. Moreover, vitamin D deficiency leads to a reduction in Ca^2+^ reuptake into the sarcoplasmic reticulum, causing a prolongation of the relaxation phase of muscle contraction, which alters muscle contractions [72].

Vitamin D is one of the most regularly used dietary supplements among athletes worldwide. While the best enhancement after vitamin D treatment occurs in athletes with critically low baseline status of 25(OH)D (under 30 ng/mL), improvements in athletic performance when the baseline is over 30 ng/mL are less expressed or even unnoticeable. In the systematic review, ergocalciferol supplementation, in contrast to cholecalciferol supplementation, was found to be ineffective at impacting muscle strength in athletes [73].

Additionally, vitamin D supplementation has proven to have a beneficial effect on muscle function in older people. For optimal fracture prevention and muscle preservation, the most optimal seems to be a serum 25-hydroxyvitamin D concentration of 60–75 nmol/L. Studies have also described reversible morphological changes in skeletal muscles of severe vitamin D-deficient patients expressing type II muscle fiber atrophy. The abovementioned changes yielded with vitamin D supplementation as vitamin D induces myogenesis and muscle protein synthesis causing an increase in the percentage of the fast twitch muscle cell (type II fibers). This type of fiber is responsible for high power output, fast muscle contraction, and muscle development. Vitamin D receptors seem to play a significant role in muscle regeneration after injury when the expression of VDR increases. A local presence and activation of 1α-hydroxylase in injured muscle fibers let the muscle synthesize an active form—1,25(OH)2D [71].

Mechanisms leading to vitamin D insufficiency in the elderly population are, among others, reduced cutaneous synthesis and exposure to sunlight, gastrointestinal malabsorption, and renal failure. Hence, because often all of the listed factors exist within critically ill patients, vitamin D supplementation presents as being rational. Although there exists limited data on the supplementation of vitamin D alone as an intervention aiming to prevent sarcopenia, oral intake of vitamin D resulting in reversing the vitamin D inadequacies may contribute to an increase in physical performance and muscle parameters. Therefore, clinicians should consider the screening of vitamin D levels in sarcopenic and sarcopenia-prone patients and advocate oral supplementation when required [74,75].

### 2.8. Anabolic-Androgenic Steroids (AAS)

The nutritional approach remains crucial in the proper recovery process but alone may be insufficient in providing enough support for ICU survivors. Therefore, exogenous administration of AASs might be recommended when considered advantageous hormonal changes are fundamental in the pathophysiology of sarcopenia. Marked reductions in gonadal steroid production are often observed in critical illness. Hypotestosteronemia is a frequent finding in critically ill patients with trauma and/or sepsis. Testosterone levels below the reference range can be present in up to 100% of mechanically ventilated individuals and might be connected with longer ICU stays [76]. It is noteworthy that in a systematic review and meta-analysis of Corona and colleagues, reduced testosterone levels resulted in up to four- and five-fold increased risk of ICU admission and death in COVID-19 patients [77]. Moreover, persistent low testosterone levels due to a catabolic state contribute to impaired recovery and rehabilitation. Thus, the introduction of interventions targeted to attenuate catabolism and muscle loss, not only during ICU stay but also after patient discharge, seems to be beneficial [78]. The AASs comprise endogenous testosterone and its pharmacology-derivated molecules. Their two primary properties, androgenic and anabolic, are accomplished due to the modulation of androgen receptor expression and interference of glucocorticoid receptor expression. Targeted anabolic testosterone analogs, in which the chemical structure of testosterone was modified, exhibit higher selectivity for muscle recovery and anabolic effects, with minimalization or almost elimination of unwanted androgenic properties. Oxandrolone and nandrolone present a myotrophic to androgenic activity ratio of 12:1 and 13:1, respectively [79]. Even though AASs are well-known for their effects on increasing muscle mass and physical function, they remain outside the first-line management of sarcopenia. The explanation for the limitation of wider use is mainly their potential adverse effects, such as an increase in blood pressure, hematocrit, and cardiovascular and prostate cancer risk. Nevertheless, these negative consequences of AASs are reported by misuse of testosterone, mainly for recreational and aesthetic purposes, and chronic supraphysiological testosterone plasma levels. In clinical controlled trials, safe AAS administration adverse effects appear significantly less [80]. In fact, in patients with preexisting stroke/cardiovascular disease and hypotestosteronemia, exogenous testosterone supplementation appears to decrease cardiovascular/stroke risk [78]. Commonly used forms of testosterone supplements in ICU include oxandrolone, nandrolone, and testosterone cypionate. Intramuscular testosterone administration promotes a 3–5 increase in muscle mass and strength when compared to transdermal testosterone [81]. Consequently, testosterone patches, because of coexisting edema and altered skin perfusion, may not be effective in adequate correction of severe testosterone deficiencies seen in critically ill patients [78]. Typical adult dosing, along with individual properties of AASs, is demonstrated in Table 1.

Supplementation of AASs should be followed by regular, weekly screening of testosterone serum concentration and liver function tests for AST/ALT [78]. While adequate testosterone correction in men targets the reference range (240–950 ng/dL) [78], there exists a lack of data in female groups, and consequently, it remains uncertain whether reference testosterone level in women (12–78 ng/dL) would be sufficient to improve physical performance. In a multi-center trial evaluating exercise and testosterone therapy in women after hip fracture, Ellen F. Binder et al. established their target on a slightly supra-physiologic serum testosterone level (110–160 ng/dL) with serum total testosterone level < 60 ng/dL as an inclusion criterion [82].

Testosterone therapy might be especially beneficial in low-T patients as in this population, adequate testosterone correction not only improves physical performance but also decreases cardiovascular/stroke risk [78]. Targeted anabolic testosterone analogs (e.g., oxandrolone, nandrolone), due to their predominant anabolic properties, might be advocated to minimize androgenic effects [78].

**Table 1:** Dosing and properties of anabolic–androgenic steroids used in ICU [78,83].

Form of Testosteron Suplement	Typical Adult Dose	Myotrophic to Androgenic Activity Ratio	Individual Properties
Oxandrolone	10 mg; twice daily; orally	13:1	- Primarily anabolic with minimal androgenic effects; - Minimal risk of liver enzyme elevation; - Effective in AIDS, COPD, and CKD patients;
Nandrolone	100–200 mg male, 50–100 mg female; intramuscularly; weekly	12:1	- Primarily anabolic with minimal androgenic effect; - Possible reduction of endogenous testosterone production;
Testosterone cypionate	200–400 mg; intramuscularly; every 2 weeks	0.7–1.3:1	- Commonly utilized outpatient testosterone replacement intervention; - More virilizing effects for women, potential aggression, and negative impact on cholesterol levels;

### 2.9. Growth Hormone, Insulin Growth Factor 1

Growth hormone is a polypeptide hormone exhibiting molecular heterogeneity. It consists of a complex mixture of molecular isoforms and their multimers [84]. GH secreted by the pituitary gland is transported in the circulation and, after binding to GH receptors, stimulates IGF-1 production in the liver. GH may also bypass the liver and bind to GH receptors in extrahepatic tissues, causing an increase in local IGF-1 production. By those pathways, GH causes metabolic and growth-promoting effects in tissues such as the bone, skeletal muscle, heart, lung, and kidney [85]. There is a piece of evidence that chronically ill patients have pathologically diminished GH secretion [86]. Additionally, circulating GH levels show a significant decline with the aging [87]. GH deficiency (GHD) is associated with alterations in metabolism, mood, and quality of life [87]. The promise surrounding the use of GH in critically ill individuals is most strongly related to its anabolic properties, specifically the protein sparring during hypercatabolism [86]. Although benefits including nitrogen retention, increased IGF-1, shortened hospital stay, and time on ventilation were observed in stable surgical, trauma, and burn injury patients, enthusiasm for the wide GH use in the critically ill population evaporated in 1999 when two large RCTs reported an association between GH therapy and increased mortality [88]. While the reason for the aforementioned finding remains not fully explained, GH replacement therapy is linked to an increase in energy expenditure, which cannot be accounted for by GH-increased lean body mass alone [89]. Another possible contributing factor might be glutamine depletion caused by the increase in protein anabolism, accelerated by an exogenous GH supplementation [88]. However, combined GH/IGF-1 therapy did not show an adverse effect on glutamine metabolism in critically ill patients [88]. Furthermore, in traumatic patients, there was no evidence of an increased mortality rate with GH/IGF-1 administration [90]. Therefore, a hypothesis has been raised that the combination therapy of GH and IGF-1 might be more advantageous and safe in catabolic patients [85]. Lower combined doses of IGF-I and GH may have additive or synergistic effects and produce the same metabolic effects as higher doses of either peptide alone, with the possibility of a reduced risk of long-term side effects. Administration of combined GH/IGF-1 therapy to patients with traumatic brain injury sustained improvement in metabolic and nutritional endpoints while avoiding uncontrolled infections and septic shock observed with higher GH doses in critically ill patients [90]. Moreover, while GH alone promotes a deterioration of glucose tolerance, the addition of IGF-1 simultaneously improves GH-induced insulin resistance [85]. The main concern regarding GH/IGF-1 therapy is the stimulation of an antiapoptotic environment in which genetically damaged cells may survive, leading to carcinogenesis [87].

Currently, there exists no sufficient data supporting GH or GH/IGF-1 therapy as a general practice in the ICU. However, the use of GH represents potential treatment or options to prevent musculoskeletal impairment, especially in ICU survivors and elderly patients after hospital discharge. A considerable number of concerns remain relating the growth hormone therapy; however, its potential benefits, in particular, while administrated along with IGF-1, should be assessed individually [85].

### 2.10. Beta-Blockers

Catecholamines are directly associated with the development of critical illness polyneuropathy, which is a component of the ICUAW mechanism [91]. Increased plasma concentration of catecholamines results in hyperdynamic circulation, endoplasmatic reticulum stress, higher basal energy expenditure, and, most importantly, catabolism of skeletal muscle [92,93]. Blocking these negative agents can potentially lead to a better outcome. Usage of propranolol during acute care, at a dose titrated to reduce heart rate by 20%, was noted to decrease cardiac workload [94]. Moreover, body composition studies have shown that propranolol diminishes skeletal muscle wasting and increases lean body mass after a major thermal injury by enhancing the intracellular recycling of free amino acids for protein synthesis. It also results in increased expression of genes involved in protein synthesis, such as heat shock proteins 70 (HSP70) [95,96]. According to available data, the most effective is to treat critically ill patients with up to 4mg/kg/day of propranolol divided into four doses per day [97,98,99]. Studies recommend combining beta-blocker therapy with oxandrolone for the best anti-CIPNM (anti-critical illness polyneuromyopathy) results [100]. In conclusion, because of the similarity of lean body mass loss mechanisms to patients with several burns, beta-blockers could play their role in the treatment of ICUAW, but more well-designed studies are required.

## 3. Conclusions

Several nutritional strategies can be learned from athletes and bodybuilders who have developed effective supplementation strategies over the last decades. Creatine and whey, as the most widely researched gym supplements, are proven to support muscle hypertrophy and might as well reduce muscle damage. HMB supplementation holds promise for preserving and increasing lean body muscle mass. Vitamin D supplementation, particularly in individuals with critically low baseline levels, shows promise to have good results. AASs may be advisable when deemed advantageous, especially considering the fundamental role of hormonal changes, such as hypotestosteronemia, in the pathophysiology of sarcopenia especially combined with B-blockers. In the case of multimodal therapy, IGF-1 offers a potential avenue for mitigating catabolic states and musculoskeletal impairment as well. An inseparable part of the recovery process, in addition to dietary and pharmaceutical intervention, is physical rehabilitation. When possible, early mobilization and resistance exercises are reported to restore rest-induced muscle loss and prevent further atrophy. With more research evaluating the possible benefit of incorporating strategies used by athletes into the recovery process of critical illness survivors, future improvements in outcomes may be noticed.

Key points:

1. Athletes and bodybuilders provide valuable insights and nutritional possibilities into effective supplementation strategies for muscle growth and regeneration, which hold potential utility in the treatment of ICU-acquired weakness due to common mechanisms of work;
2. Creatine and whey, extensively studied gym supplements, are known to promote muscle growth and potentially reduce muscle damage;
3. HMB supplementation shows promise in preserving and enhancing lean body muscle mass;
4. Vitamin D supplementation can improve outcomes, especially in individuals with critically low baseline levels;
5. Consideration of AASs may be warranted in specific cases, particularly when addressing hormonal imbalances implicated in conditions like sarcopenia combined with B-blockers.

## Abbreviations

ICUAW	Intensive Care Unit-acquired weakness
CIP	critical illness polyneuropathy
CIM	critical illness myopathy
CIPNM	critical illness polyneuromyopathy
ICU	Intensive Care Unit
MRC	Medical Research Council
MMT	Manual muscle testing
NMES	neuromuscular electrical stimulation
UPS	Ubiquitin–proteasome system
ESPEN	European Society for Clinical Nutrition and Metabolism
EN	enteral nutrition
PN	parenteral nutrition
HMB	β-Hydroxy-β-methylbutyrate
BCAA	branched-chain amino acids
WP	whey protein
VDR	Vitamin D receptors
AAS	Anabolic-androgenic steroids
Low-T	low testosterone level
IGF-1	Insulin Growth Factor 1
GH	growth hormone
